# A Simple and Accurate Method for the Determination of Related Substances in Coenzyme Q10 Soft Capsules

**DOI:** 10.3390/molecules24091767

**Published:** 2019-05-07

**Authors:** Kangde Bao, Chaojun Zhang, Shenggu Xie, Guifang Feng, Shiyu Liao, Lietao Cai, Jiajia He, Yueqin Guo, Chengxi Jiang

**Affiliations:** 1 Hangzhou Center for Biomedical Research and Innovation, School of Pharmaceutical Sciences, Wenzhou Medical University, Wenzhou 325035, China; premierGYQ@163.com; 2 Institute of Pharmacology, Zhejiang Kanglaite Pharmaceutical Co., Ltd., Hangzhou 310018, China; zhangchaojun@jemincare.com (C.Z.); fengguifang@jemincare.com (G.F.); sykirer@163.com (S.L.); 13018936379@wo.com.cn (L.C.); 3 Institute of Chemical Medicines, Zhejiang Institute for Food and Drug Control, Hangzhou 310052, China; xiesgu@163.com (S.X.); hjj1217@126.com (J.H.)

**Keywords:** determination, related substances, coenzyme Q10 soft capsules

## Abstract

As a new dosage form, coenzyme Q10 (Co-Q10) soft capsules are easily absorbed and utilized by the human body. Co-Q10 soft capsules can effectively improve the bioavailability and reduce medical costs for patients. A main concern about Co-Q10 as an active pharmaceutical ingredient (API) is how to control the total quantity of related substances. In this article, according to the degradation pattern of the API, the most easily degradable impurity (impurity X) in the sample was prepared and its chemical structure was determined. Furthermore, a simple and accurate method was developed for the determination of related substances and to avert the interference of excipient ingredients in Co-Q10 soft capsules. The approach was validated adequately and the primary impurity X was confirmed accurately. The limit of total quantity of related substances (less than 1%) could be revised to the level of specific impurity X being no more than 0.5%, in this effective quality control method of Co-Q10 soft capsules. The revised level is suggested to be included in the corresponding standard of the supplement taken from the Pharmacopoeia of the People’s Republic of China (2015 edition). This can provide a feasible method for the relevant enterprises and regulatory authorities to control the related substances of coenzyme Q10 soft capsules.

## 1. Introduction

Coenzyme Q10 (Co-Q10), which was firstly isolated from bovine myocardium in 1957, is a lipid soluble crystal named 2,3-dimethoxy-5-methyl-6-decaprenyl-1,4-benzoquinone (Ubiquinone). It is one of the commonly used clinical drugs in promoting oxidative phosphorylation, protecting the structural integrity of biofilm, treating cardiovascular diseases such as viral myocarditis, chronic cardiac insufficiency, and remedying hepatitis such as viral hepatitis, subacute liver necrosis and chronic active hepatitis [1,2,3,4]. Co-Q10 soft capsules are a new dosage form that can be easily absorbed and utilized by the human body. Obviously, Co-Q10 soft capsules effectively improve the bioavailability and reduce medical costs for patients [5].

Co-Q10 can be produced through microbial fermentation or synthesis. The homolog compound Co-Q9 and the isomer of Co-Q10 are always accompanied. It would otherwise decompose into a series of photodegradation products when exposed to light. Meanwhile, the newly introduced auxiliary materials during the preparation process have a great impact on the quantity limit of its related substances. In summary, drugs or health care products with Co-Q10 as an active pharmaceutical ingredient (API) are faced with the problem of controlling the total quantity of related substances. In relevant domestic and foreign literature and quality standards, a methodology to control the related substances of Co-Q10 soft capsules has only shown in the Pharmacopoeia of the People’s Republic of China (2015 edition) [6,7,8]. No methodology for the control of related substances can be found in the US Pharmacopoeia (USP 40 edition).

During the preparation of Co-Q10 soft capsules, the interference peak produced by the auxiliary materials had a certain influence on the determination of related substances in the quality control method. To look for the optimized method, the following were used to analyze Co-Q10 and its excipients: changing the proportion of mobile phase components, utilizing the C18 column reverse phase liquid chromatography system; utilizing the C8 column reverse phase liquid chromatography system; deploying the amino and silica gel column positive phase liquid chromatography system; employing the tetrahydrofuran and water-based reverse phase liquid chromatography system and utilizing UPC2 ultra-high performance liquid chromatography [3,9,10,11]. However, a method for the determination of related substances could not be established to control all of the impurities in Co-Q10 soft capsules, so the impurities which were easy to capture in the preparation were targeted, and the impurity most easy to degrade in the API (hereinafter referred to as impurity X) was prepared. Once the structure of impurity X was confirmed accurately and the exclusive detection method was established, the determination method for related substances in the quality specification of Co-Q10 soft capsules could be further optimized [3,9]. Because of the easy degradation characteristics of Co-Q10, the photolysis experiment to determine the main products of the degradation of Co-Q10 was employed, and the source of related substances in Co-Q10 soft capsule preparation and its influence on the total amount of related substances in the finalized product were scientifically identified. In this work, a new simple and accurate method for the control of related substances in the quality standard of Co-Q10 soft capsules was proposed.

## 2. Results and Discussion

### 2.1. Identification and Structural Analysis of the Degradable Impurity

According to the relevant determination method of the Pharmacopoeia of the People’s Republic of China (2015 edition), the auxiliary materials used in Co-Q10 soft capsules show obvious interference in the determination of the related substances in the finalized product. Co-Q10 was easily decomposed when exposed to light. Therefore, the structure of the impurity (impurity X) was investigated and the relevant determination method was established [8].

#### 2.1.1. Confirmation of Impurity X

**Figure 1:**
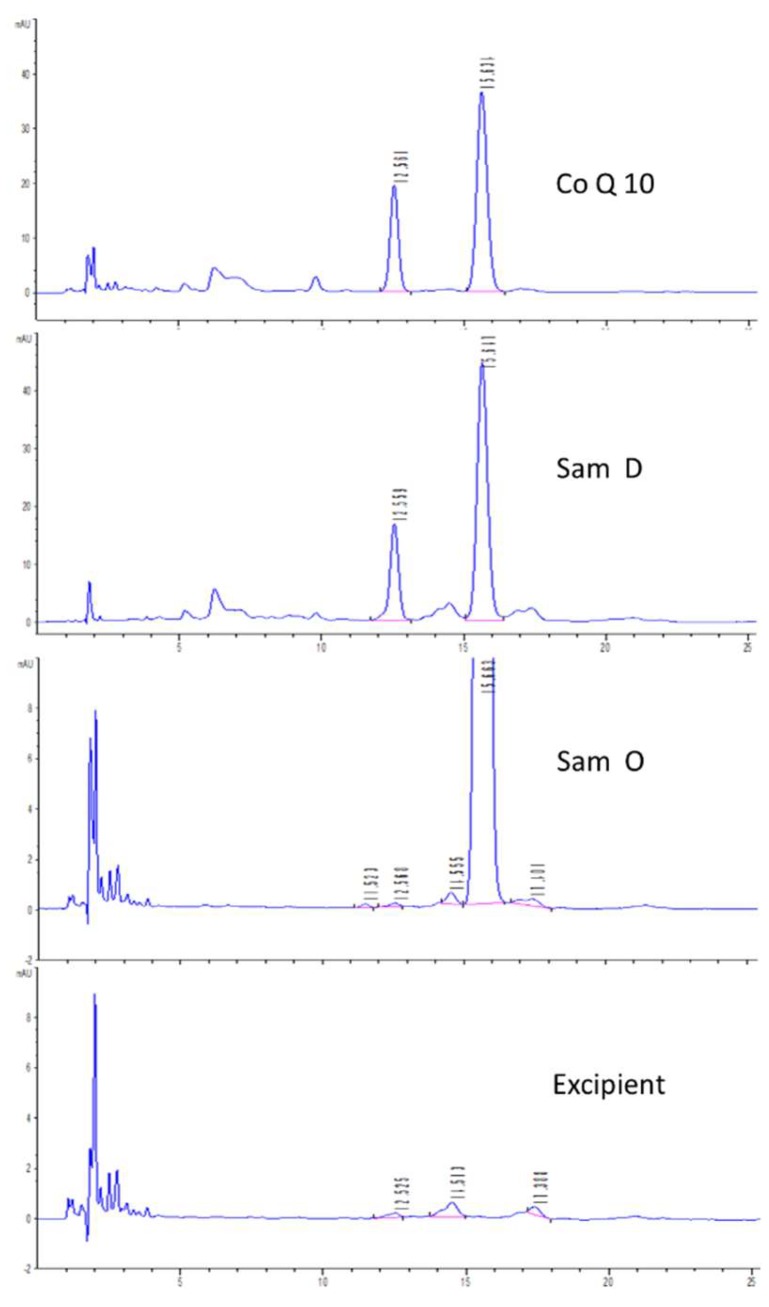
Typical chromatogram of the confirmation.

Appropriate amounts of the degradation products of Co-Q10 API and Co-Q10 soft capsules, the original Co-Q10 soft capsules and the blank excipient were detected, individually, according to the Co-Q10 test in the Pharmacopoeia of the People’s Republic of China (2015 edition) [6,9]. The results are shown in Figure 1 (from top to end: degradation Co-Q10, degradation sample, original sample and excipient).

As shown in Figure 1, Co-Q10 was easily degraded under light and high temperature, and the main degradation peak was captured at 12.5 min under the conditions of the Chinese Pharmacopoeia (2015 edition). The results showed that the content of impurity X was about 0.2%, while the content of excipients was about 0.9% in the same amount of raw materials and coenzyme Q10 soft capsules. However, this degradation may overlap with the chromatographic peaks of some components in excipients under the same conditions. Therefore, the total amount of related substances was about 1.1% (0.1% beyond the prescribed limit). The degradation peak was enriched by a pre-HPLC system, and the named impurity X was obtained for further study.

#### 2.1.2. Purity and Structure Confirmation of Impurity X

##### The Purity of Impurity X

**Figure 2:**
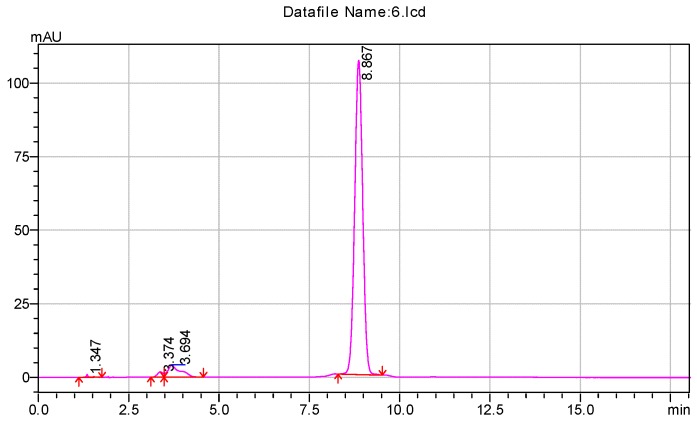
The content of impurity X detected by HPLC.

**Figure 3:**
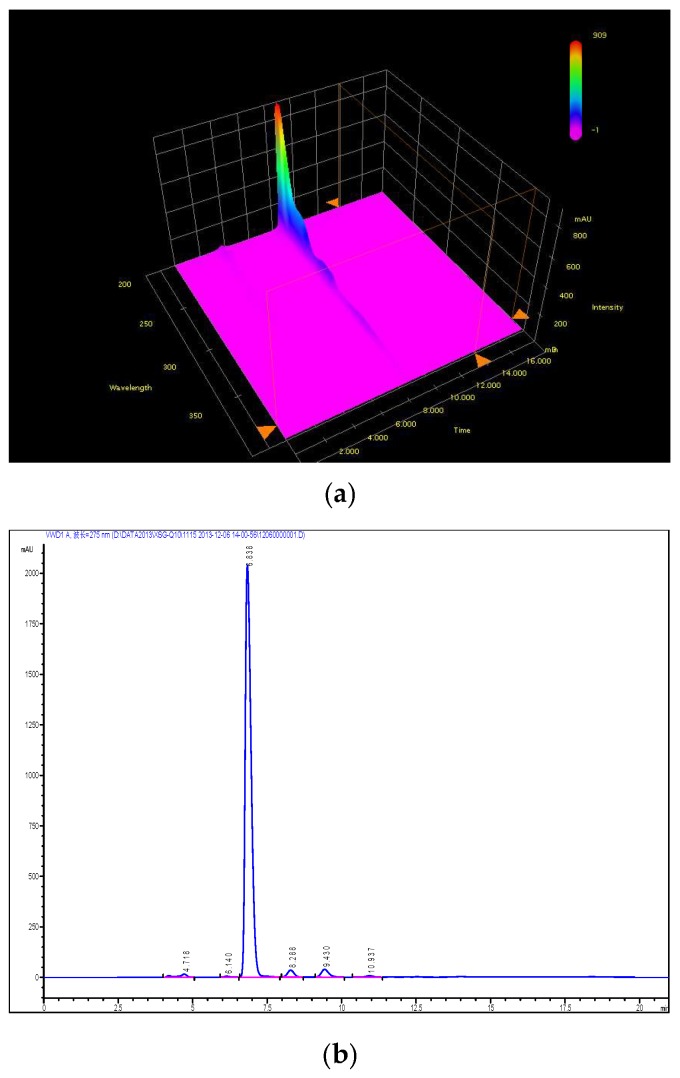
(**a**) Typical chromatograms and 3D maps of reversed-phase system for calculating impurity X content by normalization method; (**b**) Typical chromatogram of normal phase system for calculating impurity X content by normalization method.

According to the chromatographic condition and system suitability test (reversed phase system) and isomer (positive phase system) inspection methods of Co-Q10 API, combined with the general principle 0512 recorded in Chinese Pharmacopoeia (2015 edition) Part Four, impurity X was detected and its content measured at more than 93%. The results are shown in Figure 2 and Figure 3.

##### Structure Confirmation of Impurity X

**Figure 4:**
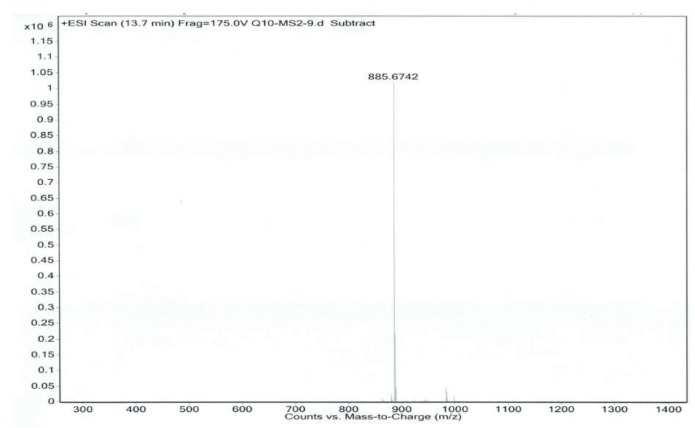
MS spectrum of Co-Q10 reference substance.

**Figure 5:**
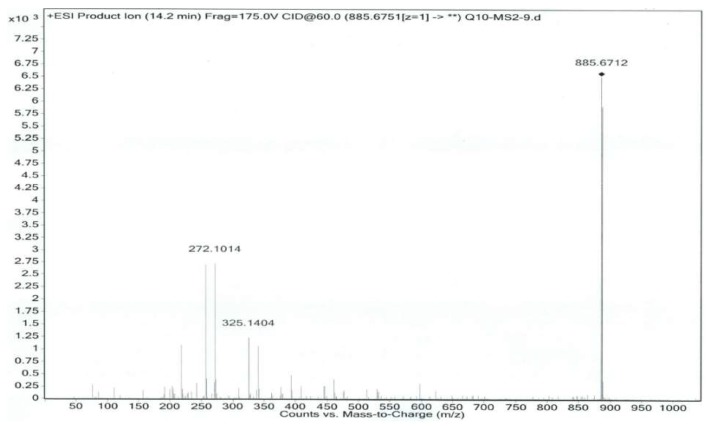
MS/MS spectrum of Co-Q10 reference substance.

**Figure 6:**
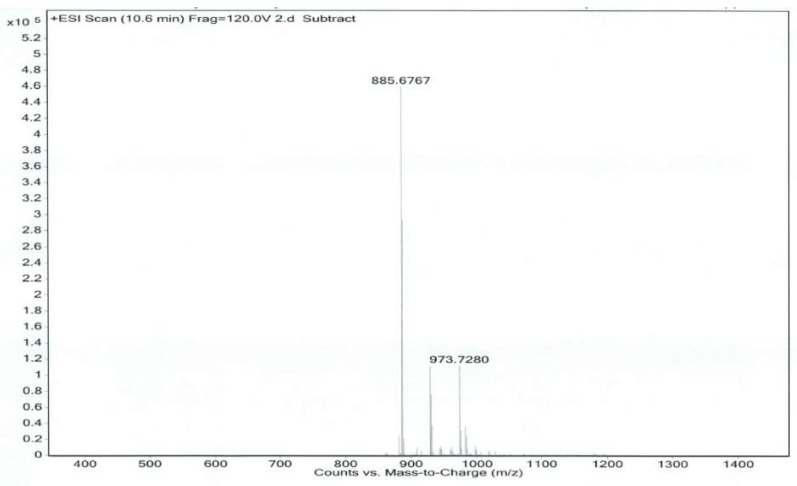
MS spectrum of impurity X.

**Figure 7:**
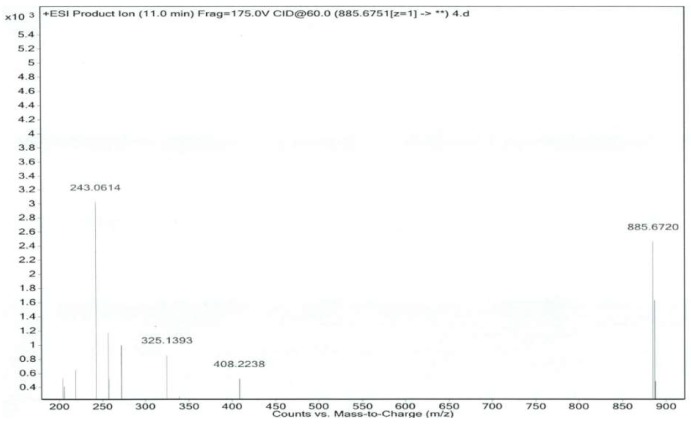
MS/MS spectrum of impurity X.

Co-Q10 reference solution and impurity X solution were precisely measured, then mass spectrometry (MS) was performed according to the chromatographic conditions above [9,10]. The MS and MS/MS (tandem mass spectrometry) of Co-Q10 reference substance are shown in Figure 4 and Figure 5. The same MS and MS/MS images of impurity X are presented in Figure 6 and Figure 7, respectively.

**Figure 8:**
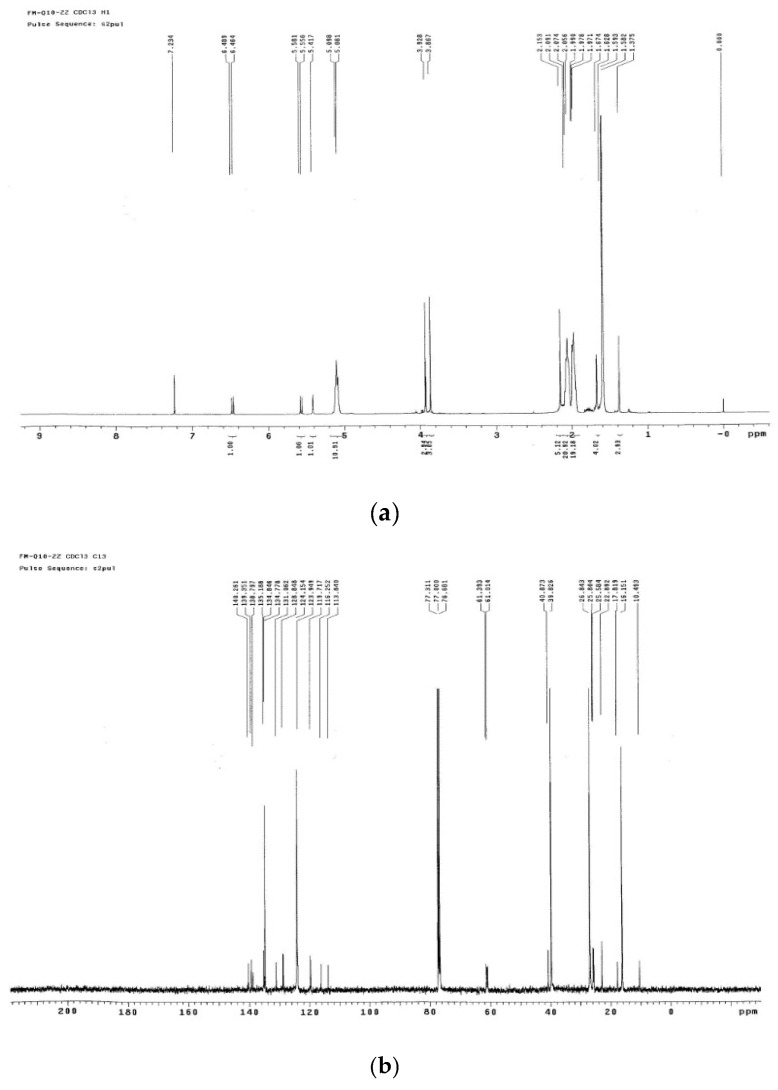

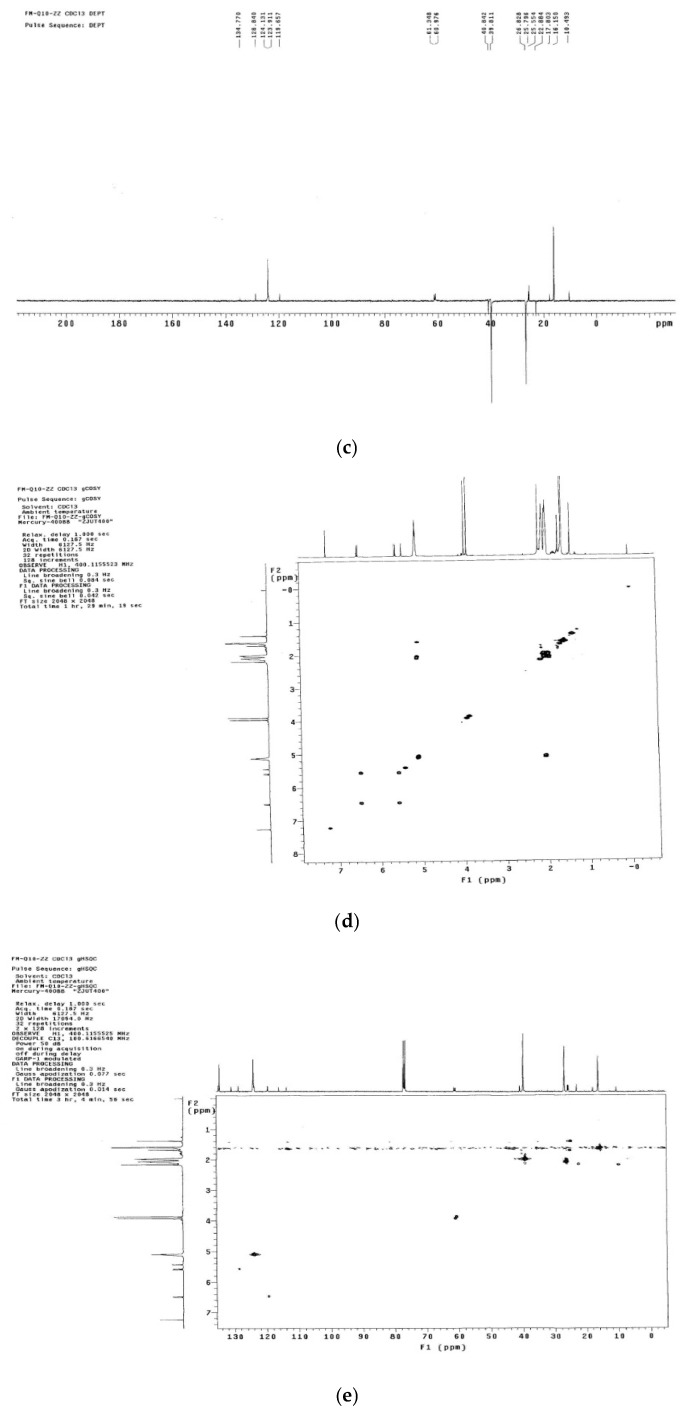

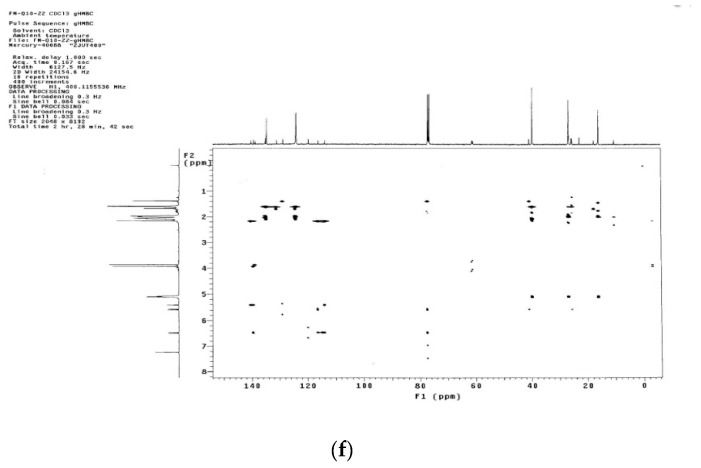
(**a**) H-NMR spectrum of impurity X; (**b**) C-NMR spectrum of impurity X; (**c**) DEPT spectrum of impurity X; (**d**) gCOSY spectrum of impurity X; (**e**) gHSQC spectrum of impurity X; (**f**) gHMBC spectrum of impurity X.

An appropriate amount of impurity X sample was taken for NMR spectrum analysis; spectra are shown in Figure 8.

**Figure 9:**
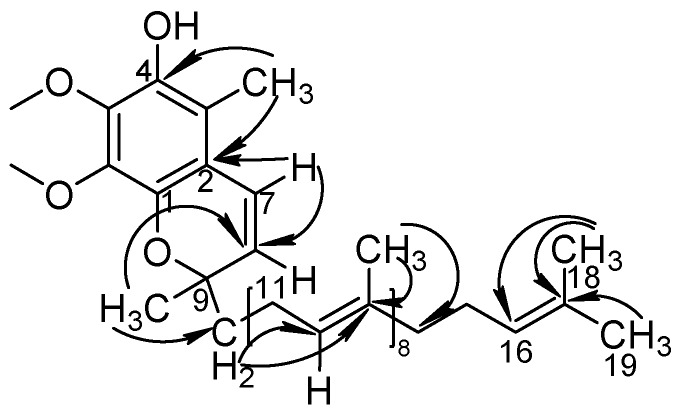
The chemical structure and key HMBC correlations of impurity X.

An [M + Na]^+^ ion with mass accuracy at *m*/*z* 885.6767 was captured by LC-QTOF-MS. The molecular formula C_59_H_90_O_4_ of this compound was deduced from ESI-MS spectrometry (calculated 862.69)—the spectra of ^1^H-NMR and ^13^C-NMR—while its unsaturation degree was 15. According to the chemical structure of Co-Q10, as well as NMR data, the compound was found to contain a long chain structure connected by multiple isopentenes end to end. Furthermore, the 160–200 signals in the carbon spectrum indicated that the para-quinoid-structure in Co-Q10 had changed. In the carbon spectrum, especially at 140.26, 139.35, 138.78, 135.19, 116.25 and 113.84, there might be a group of signals of a benzene ring. The correlation between 5.56, 5.58 (1H, *J* = 7.5 hz) and 6.46, 6.49 (1H, *J* = 7.5 hz) in the COSY spectrogram indicated that this pair of ene protons was on the same side. Combined with HSQC and HMBC spectra, the possible structure was speculated, as shown in Table 1, and impurity X was the isomer of Co-Q10 (Figure 9) [3,4,9,10,11,12,13].

### 2.2. Methodology for the Determination of Impurity X Content

Due to the complex preparation process of impurity X and the impossibility of purchasing it, the first preparation was made after referencing relevant literature and quality standards. In order to improve the operability of the quality control of Co-Q10 soft capsule preparation and avoid the problem of high cost for the preparation of reference materials, the system applicability solution preparation method was specially added to make the impurity localization method more rapid and accurate.

#### 2.2.1. Confirmation of the Preparation Conditions for the System Suitability Solution

**Figure 10:**
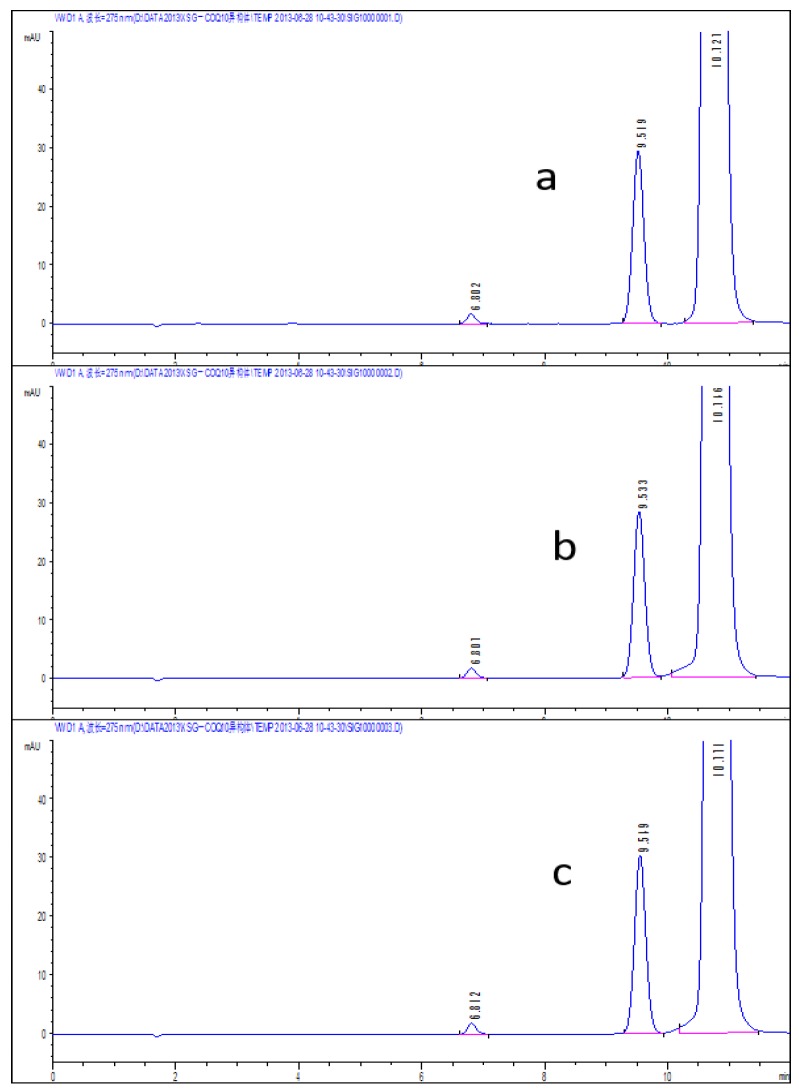
The system suitability spectrum obtained after 4 h at different concentrations of 30% hydrogen peroxide (**a** 2 μL; **b** 5 μL; **c** 10 μL).

**Figure 11:**
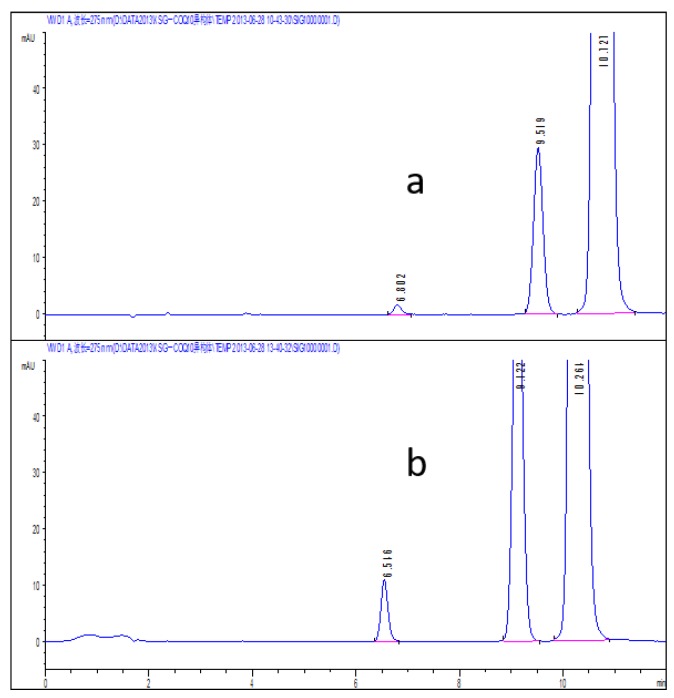
The system suitability spectrum obtained by adding 30% hydrogen peroxide solution at 2 μL for different time periods (**a** 4 h; **b** 24 h).

An amount of 10 mg of the Co-Q10 reference substance was prepared in N-hexane at a concentration of 1 mg/mL, then a 30% hydrogen peroxide solution was added at 2 μL, 5 μL and 10 μL, respectively. As the system suitability solutions, they were placed in the light box (temperature 30 ℃ and light intensity 2000 LX), respectively, for 4 h and 24 h. The results are presented in Figure 10 and Figure 11. The peaks from left to right are photodegradation impurity (impurity X), relative retention time (RRT) 0.9 isomer and Co-Q10.

It was clearly shown that the additional amount of 30% hydrogen peroxide solution did little damage to the impurities, but had more destructive influence over time. As the determination system was a positive phase system, the system suitability solution preparation condition was to add 2 μL of 30% hydrogen peroxide solution and stand for 24 h.

#### 2.2.2. Assay of the Relative Retention Time (RRT) 0.9 Chromatographic Peak 

**Figure 12:**
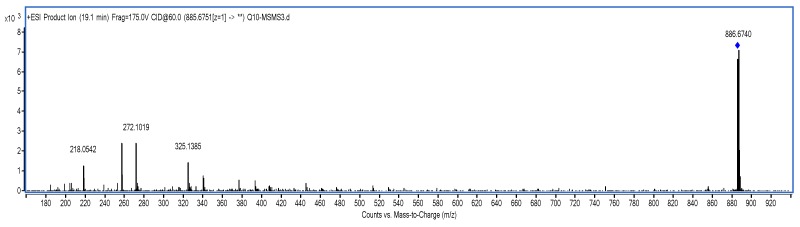
MS/MS spectrum of Co-Q10 reference substance.

**Figure 13:**
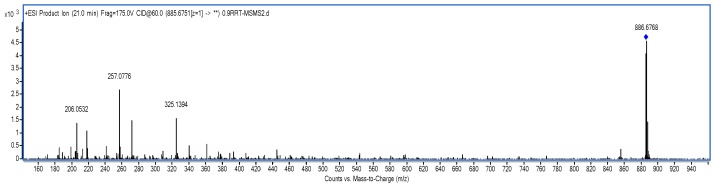
MS/MS spectrum of Co-Q10 isomer (RRT 0.9).

The RRT 0.9 peak of the system suitability solution chromatogram was assayed by Q-TOF data (Figure 12 and Figure 13), and the specific graph is summarized in Table 2:

Based on the MS and MS/MS data above, the RRT 0.9 peak was preliminarily considered as the isomer peak of Co-Q10 [9,10,11].

#### 2.2.3. Measurement of Calibration Factors 

Working solutions of Co-Q10 and impurity X, containing 0.01 mg/mL of each, were obtained by proper dilution with N-hexane.

**Figure 14:**
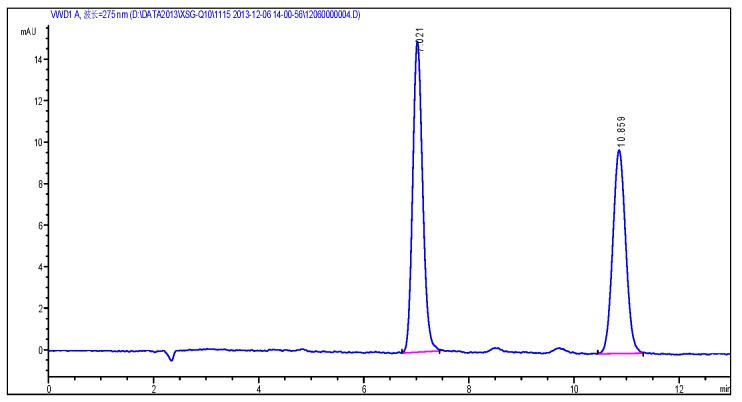
Typical chromatogram of mixed reference of impurity X (Left) and Co-Q10 (Right).

Amounts of 20 μL of solution were injected into the liquid chromatographic system. The chromatograms are recorded in Figure 14. The calculated correction factor was 1.09, while the percentage of degraded impurities was 93.2%, measured by the area normalization method (Table 3). As the degraded impurities produced by the preparation were of a small mass and oily substance, their purity and weighing errors were relatively large, and the results of the correction factor could be used as a reference [14,15,16]. 

From Table 3, it was easy to summarize that the linear equation of Co-Q10 was: y = 0.5118x + 0.9543 (R² = 0.9999), while the linear equation of impurity X was: y = 0.4705x − 0.2982 (R² = 0.9999).

#### 2.2.4. Column Robustness

**Figure 15:**
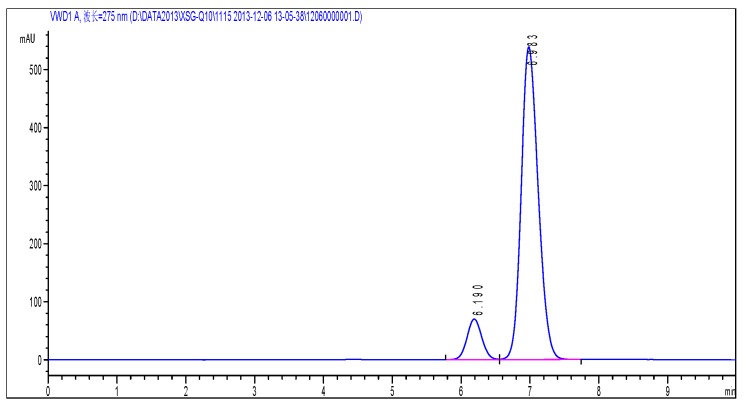
System suitability spectrum of an Agilent RX-SIL column.

The column robustness was investigated and results showed that the peak of impurity X cannot be captured by a Hypersil SiO_2_ column (250 × 4.6 mm, 5 μm). Therefore, the column type used for the quality of Co-Q10 soft capsules should be stipulated as an Agilent RX-SIL column (250 × 4.6 mm, 5 μm), with the specific spectrum shown in Figure 15 [17].

## 3. Materials and Methods 

### 3.1. Chemicals and Reagents

Co-Q10 soft capsules were supplied by Zhejiang Kanglaite Pharmaceutical Co., Ltd. (Hangzhou, China, Batch No. 20130302) and Zhejiang Hailisheng Pharmaceutical Co., Ltd. (Zhoushan, China, Batch No. 121101). Co-Q10 API were purchased from Zhejiang Xinchang Pharmaceutical Co., Ltd. (Shaoxing, China, Batch No. 1302101). Co-Q10 reference substance was obtained from National Institutes for Food and Drug Control (Beijing, China, Batch No. 140611-200803). Methanol and ethanol (HPLC grade and CP grade), as well as N-Hexane and ethyl acetate (HPLC grade), were purchased from Sinopharm Group Chemical Reagent Co. Ltd. (Shanghai, China).

### 3.2. Equipment

The HPLC detection was carried out on an Agilent 1100 liquid chromatograph system. An Agilent 1290 ultrapress liquid chromatography-mass spectrometer (Agilent, Palo Alto, CA, USA) was used for UPLC-MS/MS detections. An Agilent Prestar SD-1 liquid chromatograph system was used to prepare the subjects for the determination of impurity X. The relative molecular mass of impurity X was detected by an Agilent 6538 Q-TOF mass spectrometer, and the structure of impurity X was identified by a Varian Mercury NMR spectrometer (400 MHz, Agilent, Palo Alto, CA, USA).

### 3.3. Sample Preparation

#### 3.3.1. Preparation of Test Solution

Degradation mother liquor I and II (20 mg/mL), as well as the degradation solution I and II (1 mg/mL) of Co-Q10, were prepared by appropriate dilution with anhydrous ethanol. Then, they were put in stress conditions, temperature 40 °C and light intensity 2000 LX, for 30 days. 

Contents I and II of Co-Q10 soft capsule samples and auxiliary blank solution I and II were put in a 50 mL conical flask and dissolved with suitable anhydrous ethanol. After that, they were stored in stress conditions, temperature 40 °C and light intensity 2000 LX, for 30 days, and then metered volume (1 mg/mL) of anhydrous ethanol, respectively. 

Contents Ⅰ and Ⅱ of Co-Q10 soft capsule samples were put in a 50 mL conical flask, and metered volume (1 mg/mL) of anhydrous ethanol, respectively.

#### 3.3.2. Preparation of Impurity X

The chromatographic peak of impurity X in degradation mother liquor of Co-Q10 was prepared by an Agilent Pre-C18 column. The isocratic mobile phase was methanol–ethanol (1:1), and the flow rate was 40 mL/min. After that, the prepared liquid was decompressed and concentrated. An appropriate amount of anhydrous ethanol was added to make it dissolve, and the processes above was carried out repeatedly for secondary purification. After vacuum drying, the canary yellow liquid was sealed and kept at low temperature as the impurity X sample for further study.

#### 3.3.3. Preparation of Standard Solutions

The Co-Q10 standard solution of 0.2 mg/mL was obtained by appropriate dilution with methyl alcohol, then the solution was stored in darkness for later use.

An appropriate amount of impurity X sample with a known purity was prepared in a solution of 1.0 mg /mL, and stored as the standard solution.

### 3.4. Chromatographic Conditions

#### 3.4.1. Liquid Chromatography

The determination was carried out on an Agilent RX-SIL column (250 × 4.6 mm, 5 μm). The column temperature was kept at 25 °C. The isocratic mobile phase consisted of N-Hexane and ethyl acetate (97:3), and the flow rate was 2.0 mL/min. The detection wavelength was set at 275 nm [9,14].

The Co-Q10 degraded solution I, Co-Q10 soft capsule degraded solution I, Co-Q10 soft capsule solution I and blank auxiliary solution I were detected with injection volume 20 μL, individually. All chromatograms were recorded.

#### 3.4.2. HPLC-MS/MS 

Separation was achieved by using a Kromasil C18 column (250 × 4.6 mm, 5 μm). The column temperature was kept at 35 °C. The isocratic mobile phase consisted of methanol–ethanol (50:50), the flow rate was set at 2.0 mL/min, and the split ratio was 3:1. Electrospray ionization (ESI) source was performed at a positive ion pattern. Capillary voltage was set at 4000 V, the nebulizer pressure was 45 psig and the flow rate of dry gas was at 12 L/min [13,14,15]. 

## 4. Conclusions

A series of methods have been employed for the determination of related substances in Co-Q10 soft capsules. However, the question of how to control all impurities of Co-Q10 soft capsules is still a challenge. According to the degradation pattern of the API, the most easily degradable impurity X in the sample was prepared and its chemical structure was determined. Furthermore, a simple and accurate method was developed for the determination of related substances and to avert the interference of excipient ingredients in this article. The approach was validated adequately and the primary impurity (impurity X) was confirmed accurately. The limit of total quantity of related substances (less than 1%) could be revised to the level of specific impurity X being no more than 0.5%, in this effective quality control method of Co-Q10 soft capsules. The revised level is suggested to be included in the corresponding standard of the supplement taken from the Pharmacopoeia of the People’s Republic of China 2015. This can provide a feasible method for the relevant enterprises and regulatory authorities to control the related substances of coenzyme Q10 soft capsules.

## Figures and Tables

**Table 1:** C-attribution in the spectrogram of Impurity X.

Location	^13^C-NMR	DEPT	HSQC (Contain ^1^H-NMR)	HMBC
1	139.35	quaternary carbon	-	-
2	116.25	quaternary carbon	-	-
3	113.84	quaternary carbon	-	-
4	140.26	quaternary carbon	-	-
5	138.78	quaternary carbon	-	-
6	135.19	quaternary carbon	-	-
7	119.72	CH	6.46, 6.49 (1H, *J* = 7.5 Hz)	139.35, 116.25, 113.84
8	128.85	CH	5.56, 5.58 (1H, *J* = 7.5 Hz)	116.25, 40.87,25.58
10	40.87	CH_2_	1.97	134.85, 124.15, 26.84
11	22.89	CH_2_	2.15 (2H)	39.83, 26.84
12	124.15	CH	5.08 (9H)	39.83, 26.84,16.15
13	134.85	quaternary carbon	-	-
14	39.83	CH_2_	1.97, 1.98, 1.99 (mutiple H)	134.85, 124.15, 26.84, 16.15
15	26.84	CH_2_	2.06, 2.07, 2.09 (mutiple H)	134.85, 124.15, 123.95, 39.83
16	123.95	CH	5.10 (1H)	39.83, 26.84, 16.15
17	131.06	quaternary carbon	-	-
18	17.82	CH_3_	1.58 (3H)	131.06, 124.15
19	25.80	CH_3_	1.67 (3H)	131.06, 123.95, 17.82
20	10.49	CH_3_	2.15 (3H)	140.26, 116.25, 113.84
21	25.58	CH_3_	1.38 (3H)	128.85, 40.87, 16.15
22	16.15	CH_3_	1.59 (mutiple H)	134.85, 124.15, 39.83, 26.84
-OCH_3_	61.39	CH_3_	3.93	140.26
-OCH_3_	61.01	CH_3_	3.87	139.95

**Table 2:** Co-Q10 reference substance and impurity X primary mass spectrometry and secondary mass spectrometry data.

Item	MS	MS/MS
Coenzyme Q10	886.6740	218.0542, 257.0780, 272.1019, 325.1385
Isomer of Co-Q10	886.6768	218.0547, 257.0776, 272.1023, 325.1394

**Table 3:** Correction factor for related substances.

Peak Area of Co-Q10	Mass of Co-Q10 (ng)	Peak Area of impurity X	Mass of impurity X (ng)
220.77	431.20	262.51	560.03
167.80	323.40	198.46	420.01
111.20	215.60	131.22	280.02
55.94	107.80	65.08	140.01
28.38	53.90	32.84	70.01

## References

[1] Folkers K., Lenaz G. (1985). Coenzyme Q: Biochemistry, Bioenergetics and Clinical Applications of Ubiquinone.

[2] Crane F. (2001). Biochemical functions of coenzyme Q10. J. Am. Coll. Nutr..

[3] Zhang H., Wu Y.H. (2002). Development of the studies of coenzyme Q10. *Subvolume of Hygiene*. Forei. Med. Sci..

[4] Zhang J.S., Wang D., Huang Q.W., Wu R.J. (2009). Evaluation Studies on Related Substances of Domestic Coenzyme Q10. Northwest. Pharm. J..

[5] Littarru G.P., Tiano L. (2010). Clinical aspects of coenzyme Q10: *An. update*. Nutrition.

[6] Mu F.S., Luo M., Fu Y.J., Zhang X., Yu P., Zu Y.G. (2011). Synthesis of the Key Intermediate of Coenzyme Q10. Molecules.

[7] Contin M., Flor S., Martinefski M., Lucangioli S., Tripodi V. (2014). The use of coenzyme Q10 as a template in the development of a molecularly imprinted polymer for the selective recognition of coenzyme Q10. Anal. Chim. Acta..

[8] (2017). Pharmacopoeia of the People’s Republic of China (Part Four).

[9] Tripodi V., Flor S., Contin M., Mario C., Silvia L. (2009). Simple, highly sensitive micro HPLC method for the determination of coenzyme Q10 and its major related substances. J. Liq. Chrom. Rel. Tech^®^..

[10] Ning Y.L., W M.C., Z G. (2008). Identification of coenzyme Q10 and its impurity structure. J. Instr. Anal.

[11] Jiang P., Wu M., Zheng Y., Wang C., Li Y., Xin J., Xu G. (2004). Analysis of coenzyme Q10 in human plasma by column-switching liquid chromatography. J. Chrom. B..

[12] Turkowicz M.J., Karpińska J. (2013). Analytical problems with the determination of coenzyme Q10 in biological samples. J. Biof..

[13] Zu Y.G., Zhao C.J., Li C.Y., Zhang L. (2006). A rapid and sensitive LC-MS/MS method for determination of coenzyme Q10 in tobacco (Nicotiana tabacum L.) leaves. J. Sep. Sci..

[14] Ruiz-Jiménez J., Priego-Capote F., Mata-Granados J.M., Quesada J.M., Luque de Castro M.D. (2007). Determination of the ubiquinol-10 and ubiquinone-10 (coenzyme Q10) in human serum by liquid chromatography tandem mass spectrometry to evaluate the oxidative stress. J. Chroma. A..

[15] Rácz A., Vass A., Héberger K., Marietta F. (2015). Quantitative determination of coenzyme Q10 from dietary supplements by FT-NIR spectroscopy and statistical analysis. J. Anal. Bioanal Chem..

[16] Xue X.F., Zhao J., Chen L.Z., Zhou J.H., Yue B., Li Y., Wu L.M., Liu F.M. (2012). Analysis of coenzyme Q10 in bee pollen using online cleanup by accelerated solvent extraction and high-performance liquid chromatography. J. Food Chem..

[17] Vander H.Y., Nijhuis A., Smeyers-Verbeke J., Vandeginste B., Massart D. (2001). Guidance for robustness/ruggedness tests in method validation. J. Pharm. Biomed. Anal..

